# Risk of second primary cancer in men with breast cancer

**DOI:** 10.1186/bcr1643

**Published:** 2007-01-25

**Authors:** Sacha Satram-Hoang, Argyrios Ziogas, Hoda Anton-Culver

**Affiliations:** 1 Epidemiology Division, School of Medicine, University of California, Irvine, CA 92697, USA

## Abstract

**Introduction:**

A retrospective registry-based cohort study was conducted to examine the risk of second primary cancer following the occurrence of breast cancer in males.

**Methods:**

Data obtained from the California Cancer Registry in the period 1988 to 2003 included 1,926 men aged 85 years and younger diagnosed with a first primary breast cancer. Person-year analysis was applied to determine the risk of second primary cancers after the occurrence of a first primary breast cancer. The effects of age, race, and time since the first breast cancer diagnosis were assessed.

**Results:**

Of the 1,926 male breast cancer cases, 221 (11.5%) developed a second primary cancer. Men with first incidence of breast cancer have a significantly higher risk of second cancer (standardized incidence ratio (SIR) = 1.16, 95% confidence interval (CI) = 1.01–1.32). The risk of a second site-specific cancer is elevated for breast cancer (SIR = 52.12, 95% CI = 31.83–80.49), cutaneous melanoma (SIR = 2.98, 95% CI = 1.63–5.00) and stomach cancer (SIR = 2.11, 95% CI = 1.01–3.88). There is a general tendency towards higher risks of second malignancies among younger men compared to older men and the risk increased with the passage of time.

**Conclusion:**

Male breast cancer patients should be monitored carefully for the occurrence of second primary cancers, especially a second primary breast cancer.

## Introduction

Studying the occurrence of second primary cancers holds important implications for cancer etiology and preventive interventions. An association between a first and second cancer could indicate common host factors (genetic abnormalities, immune function and hormonal imbalances), shared environmental and/or occupational exposures, shared lifestyle factors, the effects of treatment for the first cancer [1,2], hereditary cancer syndromes [3], or even gene-environment interactions. Early age of onset of cancer, family history of disease, bilateral disease, and multiple primary cancers are hallmarks of hereditary cancer [3]. In some cases, the diagnosis of a rare cancer or cancer in the less usually affected sex, for example, male breast cancer (MBC), may also indicate hereditary cancer risk [3]. An awareness of multiple primary cancer syndromes can potentially influence the use of screening and sensitivity of screening in organs susceptible to multiple primary cancers, enabling early detection.

Three studies to date have investigated the risk of developing a second malignancy in MBC patients [4-6]. The first study evaluated the risk of subsequent breast cancer among 457 Swedish men, and reported a 93-fold excess risk for a second breast cancer [4]. Auvinen and colleagues [5] utilized the National Cancer Institute's Surveillance and Epidemiology End Results (SEER) data for 1,788 men and found a 30-fold excess risk of a second breast cancer and a 2-fold excess risk of cutaneous melanoma. The third study, by Hemminki and colleagues [6], pooled 3,409 MBC patients from 13 cancer registries around the world. An excess risk of second primary malignancy affecting the small intestine, rectum, pancreas, lymphohematopoietic system, prostate, and non-melanoma skin was elucidated [6]. The present study will attempt to confirm these findings and examine the relationships between observed multiple primaries, which may indicate a common etiology.

## Materials and methods

A registry-based cohort of men previously diagnosed with first primary breast cancer was followed through time to compare their subsequent cancer experience to the number of cancers that would be expected based on incidence rates for the general California male population. The data were obtained from the California Cancer Registry (CCR), California's statewide population-based cancer surveillance system. The CCR is a collaborative effort involving the California Department of Health Services, ten regional registries, hospitals, cancer researchers throughout the nation, and the Public Health Institute. It collects information about all cancers diagnosed in California (except basal and squamous cell carcinoma of the skin and carcinoma *in situ *of the cervix). The database includes information on demographics, tumor characteristics (stage, grade, histology, laterality, behavior, and hormone receptor status), treatment, survival, and reporting hospital type.

The present study includes 1,926 males aged 85 years or less diagnosed with first primary breast cancer between 1 January 1988 and 31 October 2003, registered at the CCR. A second primary cancer was defined in this study as a malignant metachronous tumor that developed at least two months after first primary male breast cancer diagnosis. There were 221 (11.5%) second primaries diagnosed in this cohort of men during the study period. Synchronous tumors occurring less than two months from first primary breast cancer were excluded in order to control for detection bias due to diagnostic methodology and also to be consistent with prior research conducted in the same geographic region [5]. Ninety-three percent of the cohort received surgery. In addition, 25% underwent adjuvant hormone therapy, 24% chemotherapy, and 20%, radiation therapy, while 19% of the cohort had some combination therapy. No men were excluded from the analysis due to missing data as information regarding second malignancy, date of diagnosis and date of last contact or death were available for all patients.

For the purposes of rate adjustment, five-year age groups, resulting in 18 age categories, were used in calculating the expected number of cases. Race/ethnicity was categorized into five mutually exclusive groups: non-Hispanic white, Black, Hispanic, Asian/Pacific Islander, and other/unknown. To calculate the expected number of cancers for the other/unknown group, we used the incidence rates of all others combined. The calendar period was classified in five-year categories of diagnosis: 1988 to 1992, 1993 to 1997, and 1998 to 2003. Tumor site codes in accordance with SEER definitions for breast, prostate, colorectal, lung and bronchus, bladder, melanoma, and stomach cancer were obtained from the CCR [7]. The unit of analysis in our study was person/days at risk in order to maximize use of fractions of years if applicable. Person-years of observation were calculated two months from the date of breast cancer diagnosis up to and including age 85, the date of the second primary cancer, date of last contact or death, or study end (31 December 2003), whichever came first.

### Statistical analysis

All statistical analysis conducted utilized SAS v 9.1 software. (SAS Institute Inc., SAS Campus Drive, Cary, North Carolina 27513, USA) Using the frequency procedure in SAS, the distribution of risk factors of interest among cohort members (Table 1) was evaluated. Chi-square analysis for categorical data and *t*-test for continuous data were used to compare the characteristics between patients who subsequently developed a second primary to those who did not (Table 1). Differences with a probability of *p *< 0.05 were considered statistically significant.

Person-years analysis, adjusted for age, race, calendar period, and tumor site, was used to calculate the standardized incidence ratio (SIR) (Tables 2 to 5). The SIR is a comparison of the incidence of second primary tumors among study patients (observed) with the incidence of the same tumors in the general population of California males (expected). Calculation of the expected numbers of second primaries was accomplished by applying sex, five-year age, race, calendar period and site-specific incidence rates from the general population of California males to the person-years at risk in the study population. To assess statistical significance, exact 95% confidence intervals (CI) were computed around the SIR assuming that the observed number of second cancers followed a Poisson distribution.

## Results

The characteristics of men diagnosed with first primary breast cancer are shown in Table 1. The cohort contained a total of 1,926 men of which 221 (11.47%) subsequently developed a second metachronous malignancy. Approximately 68% of the cohort were 60 years of age or older. Patients who developed a second cancer were older (mean age = 66.90) compared to patients who did not experience a subsequent cancer diagnosis (mean age = 64.16) (p = 0.0002). The mean and median follow-up times for this cohort were 4.39 and 3.36 years, respectively, with no overall significant difference in follow-up time between the two groups. The majority of cases were non-Hispanic white (76%) with all other races (blacks, Hispanics, Asians and other/unknown) making up the remainder of the population.

The overall SIRs for second malignancy by tumor site, controlling for age, race and calendar period, are shown in Table 2 for sites where at least ten cases were recorded. The observed number of second primary malignancies was 221 compared to 190.42 expected, thereby producing an SIR of 1.16 (95% CI = 1.01–1.32), which shows an excess risk of 16%. The excess risk of subsequent cancer decreased to 5% when second breast cancer was excluded and the result is not statistically significant. Overall, a significant excess of second primary was noted for breast cancer (SIR = 52.12, 95% CI = 31.83–80.49), cutaneous melanoma (SIR = 2.98, 95% CI = 1.63–5.00), and stomach cancer (SIR = 2.11, 95% CI = 1.01–3.88).

The effect of diagnostic age on risk of subsequent cancer is presented in Table 3. The SIR or risk ratio for all malignancies combined (breast included and breast excluded) was highest in the younger population. A similar pattern was also observed for second breast cancer with higher excess risks starting in the <60 years age group and decreasing with increasing age. A significant excess of skin melanoma (SIR = 3.18, 95% CI = 1.17–6.93) was noted among men 70 years or older. In patients diagnosed between 60 and 69 years, a 2-fold increased risk of colorectal cancer was observed. Patients 70 years or older experienced a lower risk of lung and bronchus cancer (SIR = 0.47, 95% CI = 0.19–0.97).

The effect of race on subsequent cancer risk, controlling for age and calendar period, is shown in Table 4. The 39 observed second primaries in the 'other' race/ethnic category comprise 15 black, 17 Hispanic and 7 Asian/Pacific Island men. Overall, non-Hispanic whites (SIR = 1.19, 95% CI = 1.02–1.38) showed a significant excess of subsequent cancers. When breast was excluded, the excess of second primaries was no longer observed. Second breast cancer is elevated in both racial/ethnic categories. However, the excess risk was more prominent in the 'other' ethnic background compared to non-Hispanic whites. The increased risk of second primary breast cancer among the 'other' racial/ethnic category is largely due to the risk among blacks. The risk of melanoma among non-Hispanic white men was three-fold that expected. A significant excess in stomach cancer was observed in non-Hispanic white males (SIR = 2.53, 95% CI = 1.09–4.99).

The SIRs for second malignancy by length of time after breast cancer diagnosis, controlling for age, race and calendar period, is shown in Table 5. The risk of all malignancies combined was significantly lower than expected in the first year of follow-up (SIR = 0.74, 95% CI = 0.53–0.99) and higher than expected after 5 years of follow-up (SIR = 1.63, 95% CI = 1.31–2.00). When breast cancer was excluded, the risk was 21% lower than expected in the first 5 years after diagnosis, but later increased to 45% after 5 years of follow-up. Second breast cancer was higher in each follow-up category, with the highest risk after five years of follow-up. A higher SIR was observed after 5 years from diagnosis for bladder cancer (SIR = 2.89, 95% CI = 1.49–5.05). Melanoma was in excess for 2 follow-up periods: 1 to 5 years (SIR = 2.68, 95% CI = 1.08–5.53) and >5 years (SIR = 4.15, 95% CI = 1.52–9.02). Lung and bronchus cancer was protective in the first five years after diagnosis. We also analyzed the data stratified by treatment type and subsequent cancer risk. We did not find any statistical associations with the risk of second cancers.

After more than five years had elapsed, significant excess risks were associated with men in the younger age categories (Table 5). The risk was 3-fold in men under 60 and 2-fold in men 60 to 69 years of age at diagnosis. A 62% lower risk was noted in men 60 to 69 years of age within the first year of follow-up time.

## Discussion

Our study shows that men diagnosed with a first primary breast cancer have a 16% increased risk of developing a new primary cancer in comparison with men in the general population. The prior US study, based on the geographic areas included in the SEER program, did not find an overall excess of second primary cancer [5]. That study accounted for roughly 50% of California.

Breast cancers in men are characteristic of the *BRCA2 *phenotype, which accounts for approximately 15% of all MBC [8]. Founder *BRCA2 *mutations contribute even higher prevalence rates of MBC in countries like Iceland, Sweden, Hungary, and also in Ashkenazi Jewish populations [8]. The high risks for the presence of bilateral breast disease or multiple disease sites in one organ, especially among younger men, in the current investigation as well as in prior investigations are a likely indicator of a hereditary cancer syndrome [3,9].

Male tumors related to *BRCA1 *and *BRCA2 *include breast, melanoma, stomach, prostate, colon and pancreatic cancer [8]. The risk is even more pronounced among carriers younger than 65 for melanoma, stomach, and prostate cancer [10,11]. Although in MBC a second breast cancer has the strongest association, melanoma was in excess consistently throughout the analysis and also appears to have a high relative risk, confirmed by the prior US investigation [5], but uncorroborated by Hemminki and colleagues [6]. The excess risk of melanoma was more prominent among older men, in non-Hispanic whites, and after one year of follow-up. Epidemiological studies on malignant melanoma and breast cancer suggest the influence of steroid hormones on the etiology and development of these tumors. Melanoma and breast cancer are more common in women than men, implicating the role of female hormones and further strengthening the argument of a common etiological pathway between the two. A high socio-economic status is another shared risk factor [12], but this may represent a proxy for race, as non-Hispanic whites have higher rates of both diseases. In regards to shared genetic susceptibility, both the *CDKN2A *mutation and the *BRCA2 *mutation are linked. Families with the *CDKN2A *mutation have an increased risk not only of multiple melanomas and pancreatic carcinoma but also of breast cancer [13,14]. Further, an excess of melanoma has been reported in 173 *BRCA2 *families, particularly among carriers younger than 65 [10].

We observed an overall excess of stomach cancer, a result inconsistent in the two prior investigations [5,6]. Obesity is one of the main risk factors for gastric cancer [15,16] and MBC [17-21] and this common risk factor may, to some degree, explain the relationship between the two.

The risk of lung cancer was lower than expected for men ≥70 years of age during the first 5 years of follow-up after their breast cancer. The observed lower number of lung cancer cases may be an underestimate due to potential competing risk of dying due to other conditions.

We examined treatment effects (not shown) on risk of subsequent cancer. There was no statistical association of subsequent cancer risk with prior hormone therapy, radiation therapy, or chemotherapy on risk with the exception of second breast cancer. The increased SIR for second primary breast cancer in patients with and without prior hormone therapy and chemotherapy is more likely to be related to the rarity of breast cancer in men, which causes each observed case to contribute a substantial portion to the risk estimate. In addition, the increased risk of second primary breast cancer observed with a five year latency period may be attributable to advances in breast cancer diagnosis and treatment, which improve prognosis, and thus patients may live longer and are at a higher risk for second primary breast cancer.

The high rate of subsequent cancer among non-Hispanic whites may be due to better follow-up, better treatment or they may have a survival advantage to develop a second cancer. This is exemplified by the higher mean length of survival adjusted for age observed in non-Hispanic whites compared to other racial/ethnic groups. Based on time to death after breast cancer diagnosis, non-Hispanic whites experienced the highest mean survival of 4.49 years (*n *= 1,454), followed by 4.38 for Asian/Pacific Islanders (*n *= 107), 4.14 for blacks (*n *= 157) and 3.86 for Hispanics (*n *= 170) in this study. The excess of stomach cancer among non-Hispanic white males is unexpected as it is usually more prevalent in other racial/ethnic groups, particularly Asian/Pacific Islanders [22].

The overall risk of subsequent malignancies other than breast cancer was not significantly elevated, confirming the observations reported by Hemminki and colleagues [6]. The differences in risk estimates between investigations could be explained by several factors. First, there are important distinctions in racial/ethnic distribution between the Swedish populations (mostly Caucasian) and the diverse California population that may account for the discrepancy. Second, differences in screening practices, quality of healthcare, and access to and use of healthcare between the populations may also play a role. Thirdly, the variation of dietary, lifestyle and environment exposures, as well as genomic reactions to environmental exposures may play a role in the differences observed.

## Strengths and limitations

A strength of the present study is that it is a registry-based analysis including a large number of cases of a rare cancer such as MBC. The data are of high quality as they are carefully controlled by the well-recognized, statewide, population-based cancer registry in California. Because only one registry was used in this analysis the quality of data is consistent over time. Additionally, confounding variables such as age and race were controlled-for, while other studies did not control for the effects of race [5,6]. This could explain some of the contradictory results between investigations, making the findings of the present study more meaningful.

The limitations of this study include the unavailable data on potentially confounding factors such as family history, genetic predisposition and environmental exposures. Also, about one-third of those that were diagnosed after 1998 were followed for less than five years, which may be insufficient time to record subsequent primaries as cancer typically has a long latency period. Conversely, because this cohort consists of cancer patients, they are more likely to be diagnosed with a second cancer due to increased patient awareness and increased surveillance compared to men in the general population. It is possible that the second primary breast cancers after first primary breast cancer may be misdiagnosed metastases. However, this is unlikely as we have excluded all tumors that do not follow the SEER guidelines for multiple primaries. We conducted a large number of comparisons and, therefore, we recognize that some of the results might be due to chance.

## Conclusion

Our retrospective cohort study shows that MBC patients have a 16% higher risk of developing a second primary cancer than men in the general population, and the risk is more apparent in younger men. These results will have substantial medical implications for cancer patients as well as for relatives that are at risk of the disease. Medical interventions to address increased risk of second cancer include increased surveillance, chemoprevention, and prophylactic surgery. Future investigations should further explore the role of adjuvant hormonal, radiation, and chemotherapy with a longer follow-up period and larger patient population to better describe the risk involved. Other possible areas of future investigation could examine the risk of MBC after first primary melanoma and stomach cancer.

## Abbreviations

CCR = California Cancer Registry; CI = confidence interval; MBC = male breast cancer; SIR = standardized incidence ratio.

## Competing interests

The authors declare that they have no competing interests.

## Authors' contributions

SS designed and carried out the data analysis and drafted the manuscript. AZ helped guide the statistical analysis and edit the manuscript. HAC participated in the design of the study and helped edit the manuscript. All authors read and approved the final manuscript.

## Figures and Tables

**Table 1:** Characteristics of male breast cancer patients

Characteristic	No second cancer	Second cancer	Total	*P *value
Age (mean years (SD))	64.16 (12.90)	66.90 (9.72)	64.47 (12.61)	0.0002
Follow-up (mean years (SD))	4.39 (3.74)	4.38 (3.31)	4.39 (3.69)	0.9405
Age (median years (IQR)	66.00 (55.00–74.00)	68.00 (61.00–73.00)	66.00 (56.00–74.00)	
Follow-up (median years (IQR)	3.35 (1.33–6.69)	3.39 (1.46–6.82)	3.36 (1.33–6.72)	
Age (no. of subjects (percent))				
<60 years	577 (33.84)	41 (18.55)	618 (32.09)	<0.0001
60 to 69 years	458 (26.86)	89 (40.27)	547 (28.04)	
70+ years	670 (39.30)	91 (41.18)	761 (39.51)	
Follow-up (no. of subjects (percent))				
<1 year	342 (20.06)	42 (19.00)	384 (19.94)	0.4921
1 to 5 years	731 (42.87)	88 (39.82)	819 (42.52)	
>5 years	632 (37.07)	91 (41.18)	723 (37.54)	
Race (no. of subjects (percent))				
Non-Hispanic white	1,272 (74.60)	182 (82.35)	1,454 (75.49)	0.0117
Other	433 (25.40)	39 (17.65)	472 (24.51)	
Total (no. of subjects (percent))	1,705 (88.53)	221 (11.47)	1,926 (100.00)	

A *p *value < 0.05 means statistically significant. IQR, interquartile range; SD, standard deviation.

**Table 2:** Risk of second primary cancer among men with first primary breast cancer

Cancer site	N	PERYR	OBS	EXP	SIR (95 percent CI)
All malignancies, breast included	1,926	8,460.08	221	190.42	**1.16 **(1.01–1.32)
All malignancies, breast excluded	1,926	8,529.63	201	191.63	1.05 (0.91–1.20)
Prostate	1,926	8,803.74	69	72.30	0.95(0.74–1.21)
Second breast	1,906	8,947.89	20	0.38	**52.12 **(31.83–80.49)
Colorectal	1,926	9,025.49	26	22.64	1.15 (0.75–1.68)
Lung and bronchus	1,926	9,065.75	23	32.76	0.70 (0.45–1.05)
Bladder	1,926	9,031.02	20	13.46	1.49 (0.91–2.29)
Melanoma^a^	1,454	6,971.11	14	4.69	**2.98 **(1.63–5.00)
Stomach	1,926	9,074.43	10	4.74	**2.11 **(1.01–3.88)

Bold numbers indicate that the 95 percent confidence interval (CI) does not include 1.00. ^a^Non-Hispanic whites only. EXP, expected; N, number of subjects; OBS, observed; PERYR, person years at risk; SIR, standardized incidence ratios.

**Table 3:** Risk of second primary cancer by age at first primary male breast cancer diagnosis

	Age (years)														
Cancer site	<60					60 to 69					≥70				

	N	PERYR	OBS	EXP	SIR (95 percent CI)	N	PERYR	OBS	EXP	SIR (95 percent CI)	N	PERYR	OBS	EXP	SIR (95 percent CI)

All, breast included	618	2,912.85	41	28.08	**1.42 **(1.02–1.94)	547	2,681.23	89	72.35	1.24 (1.00–1.53)	761	2,866.01	91	89.99	1.01 (0.81–1.24)
All, breast excluded	618	2,929.25	36	28.29	1.24 (0.86–1.72)	547	2,716.56	80	73.08	1.11 (0.88–1.38)	761	2,883.83	85	90.26	0.94 (0.75–1.16)
Prostate	618	2,982.99	15	8.41	1.78 (1.00–2.94)	547	2,826.47	27	29.32	0.92 (0.61–1.34)	761	2,994.29	27	34.56	0.78 (0.51–1.14)
Second breast	605	2,992.73	5	0.06	**81.38 **(26.42–189.92)	543	2,871.22	9	0.14	**64.23 **(29.37–121.92)	758	3,083.94	6	0.18	**32.98 **(12.09–71.68)
Colorectal	618	3,047.87	3	3.11	0.96 (0.20–2.82)	547	2,891.37	15	8.19	**1.83 **(1.03–3.02)	761	3,086.25	8	11.34	0.71 (0.30–1.39)
Lung and bronchus	618	3,053.53	3	5.15	0.58 (0.12–1.70)	547	2,902.69	13	12.81	1.02 (0.54–1.74)	761	3,109.52	7	14.80	**0.47 **(0.19–0.97)
Bladder	618	3,041.90	3	1.62	1.85 (0.38–5.41)	547	2,901.04	6	4.87	1.23 (0.45–2.68)	761	3,088.07	11	6.97	1.58 (0.79–2.82)
Melanoma^a^	419	2,166.33	3	1.09	2.74 (0.57–8.01)	427	2,358.83	5	1.72	2.92 (0.95–6.80)	686	2,445.95	6	1.89	**3.18 **(1.17–6.93)
Stomach	618	3,054.70	0	0.66	0.00 (0.00–4.54)	547	2,909.55	4	1.66	2.41 (0.66–6.16)	761	3,110.18	6	2.42	2.48 (0.91–5.40)

Bold numbers indicate that the 95 percent confidence interval (CI) does not include 1.00. ^a^Non-Hispanic whites only. EXP, expected; N, number of subjects; OBS, observed; PERYR, person years at risk; SIR, standardized incidence ratios.

**Table 4:** Risk of second primary cancer after first primary male breast cancer diagnosis by race

	Race									
	Non-Hispanic white					Other				

Cancer Site	N	PERYR	OBS	EXP	SIR (95 percent CI)	N	PERYR	OBS	EXP	SIR (95 percent CI)

All, breast included	1,454	6,523.25	182	153.02	**1.19 **(1.02–1.38)	472	1,936.84	39	37.40	1.04 (0.74–1.43)
All, breast excluded	1,454	6,572.88	168	153.92	1.09 (0.93–1.27)	472	1,956.75	33	37.71	0.88 (0.60–1.23)
Prostate	1,454	6,801.52	55	57.34	0.96 (0.72–1.25)	472	2,002.21	14	14.96	0.94 (0.51–1.57)
Second breast	1,437	6,908.47	14	0.31	**44.73 **(24.45–75.04)	469	2,039.42	6	0.07	**84.83 **(31.13–184.63)
Colorectal	1,454	6,976.41	22	18.32	1.20 (0.75–1.82)	472	2,049.09	4	4.32	0.93 (0.25–2.37)
Lung and bronchus	1,454	6,999.32	19	26.40	0.72 (0.43–1.12)	472	2,066.43	4	6.36	0.63 (0.17–1.61)
Bladder	1,454	6,963.65	19	11.93	1.59 (0.96–2.49)	472	2,067.37	1	1.53	0.65 (0.02–3.65)
Melanoma^a^	1,454	6,971.11	14	4.69	**2.98 **(1.63–5.00)	-	-	-	-	-
Stomach	1,454	7,013.32	8	3.16	**2.53 **(1.09–4.99)	472	2,061.12	2	1.58	1.27 (0.15–4.57)

Bold numbers indicate that the 95 percent confidence interval (CI) does not include 1.00. ^a^Non-Hispanic whites only. EXP, expected; N, number of subjects; OBS, observed; PERYR, person years at risk; SIR, standardized incidence ratios.

**Table 5:** Risk of second primary cancer by time after first primary male breast cancer

	<1 year					1 to 5 years					>5 years				
Cancer site	N	PERYR	OBS	EXP	SIR (95 percent CI)	N	PERYR	OBS	EXP	SIR (95 percent CI)	N	PERYR	OBS	EXP	SIR (95 percent CI)

All, breast included	1,926	1,653.87	42	57.12	**0.74 **(0.53–0.99)	1,884	4332.66	88	101.51	0.87 (0.70–1.07)	1,796	2,469.34	91	55.92	**1.63 **(1.31–2.00)
All, breast excluded	1,926	1,655.89	38	57.09	**0.67 **(0.47–0.91)	1,888	4359.60	81	101.92	**0.79 **(0.63–0.99)	1,807	2,509.90	82	56.64	**1.45 **(1.15–1.80)
Prostate	1,926	1,666.28	14	20.83	0.67 (0.37–1.13)	1,912	4450.10	27	37.81	0.71 (0.47–1.04)	1,885	2,683.08	28	21.89	1.28 (0.85–1.85)
Second breast	1,926	1,654.10	4	0.11	**34.90 **(9.51–89.35)	1,922	4478.09	7	0.21	**33.77 **(13.58–69.57)	1,915	2,811.43	9	0.12	**75.85 **(34.68–143.99)
Colorectal	1,926	1,670.38	5	6.23	0.80 (0.26–1.87)	1,921	4524.94	11	11.66	0.94 (0.47–1.69)	1,910	2,825.87	10	7.11	1.41 (0.67–2.59)
Lung and bronchus	1,926	1,672.78	4	9.23	0.43 (0.12–1.11)	1,922	4540.41	6	17.21	**0.35 **(0.13–0.76)	1,916	2,848.25	13	10.82	1.20 (0.64–2.05)
Bladder	1,926	1,671.12	4	3.73	1.07 (0.29–2.74)	1,922	4534.16	4	6.97	0.57 (0.16–1.47)	1,918	2,821.43	12	4.15	**2.89 **(1.49–5.05)
Melanoma^a^	1,454	1,276.50	1	1.48	0.68 (0.02–3.77)	1,453	3491.48	7	2.61	**2.68 **(1.08–5.53)	1,446	2,199.81	6	1.45	**4.15 **(1.52–9.02)
Stomach	1,926	1,672.98	2	1.30	1.54 (0.19–5.58)	1,924	4545.53	4	2.43	1.65 (0.45–4.21)	1,920	2,851.61	4	1.50	2.66 (0.72–6.81)
Age (years)															
<60	618	531.05	6	5.85	1.02 (0.38–2.23)	612	1445.16	12	12.41	0.89 (0.44–1.59)	600	935.26	23	8.18	**2.81 **(1.78–4.22)
60 to 69	547	491.85	7	18.38	**0.38 **(0.15–0.78)	540	1329.22	36	35.41	1.04 (0.74–1.44)	504	858.89	46	23.64	**1.95 **(1.42–2.60)
70+	761	630.97	29	32.89	0.88 (0.59–1.27)	732	1558.27	40	53.69	0.74 (0.53–1.01)	692	675.19	22	24.10	0.91 (0.57–1.38)

Bold numbers indicate that the 95 percent confidence interval (CI) does not include 1.00. ^a^Non-Hispanic whites only. EXP, expected; N, number of subjects; OBS, observed; PERYR, person years at risk; SIR, standardized incidence ratios.
